# Relationship between smoking and pulmonary tuberculosis based on positive sputum smears

**DOI:** 10.3205/dgkh000513

**Published:** 2024-11-05

**Authors:** Mahesh Shinde, Sangramsingh Dixit, Mihir Patel, Atharva Sharma, Juily Satam, Yogeshwari Patil, Dheer Upadhyay, Shreyasi Chiwadshetti, Adhiraj Mathur, Varad Rege, Arunojya Kumari, Hiya Seth

**Affiliations:** 1 HBT Medical College and Dr. RN Cooper Hospital Juhu, Mumbai, India

**Keywords:** smoking, pulmonary tuberculosis, smoking index, National Tuberculosis Control Program, directly observed treatment, DOTS

## Abstract

**Introduction:**

Smoking and tuberculosis are the two major, global health problems. Not only active smokers but also passive smokers are at risk of becoming infected with tuberculosis. Through many mechanisms, smoking decreases immunity and predisposes to numerous infections. This has a negative impact on our health system. This knowledge of the association between smoking and tuberculosis can be utilized to develop a program for TB prevention and control.

**Method:**

This is a retrospective observational study carried out over a period of 18 months on 100 diagnosed cases of sputum smear-positive pulmonary tuberculosis patients undergoing treatment at Mumbai Maharashtra India as a part of the Revised National Tuberculosis Control Program (RNTCP).

**Results:**

1+ sputum positivity was observed in a higher number of nonsmokers (77%) followed by ex-smokers (43%) and smokers (2%), 2+ and 3+ sputum positivity was observed in a higher number of smokers (63% and 35%, respectively) followed by ex-smokers (29%) and nonsmokers (18% and 5%, respectively).

**Conclusion:**

Smokers demonstrated extensive infiltrates as compared to nonsmokers. Additionally, as the severity of smoking increased (smoking index), and the bacterial load also increased (higher grades of sputum positivity). Smokers also had poorer treatment outcomes than did nonsmokers.

## Zusammenfassung

**Einleitung:**

Rauchen und Tuberkulose sind die beiden größten Gesundheitsprobleme in der Welt. Nicht nur aktive Raucher, sondern auch Passivraucher sind dem Risiko ausgesetzt, an Tuberkulose zu erkranken. Rauchen schwächt auf vielfältige Weise das Immunsystem und erhöht die Anfälligkeit für Infektionen. Das hat negative Auswirkungen auf das Gesundheitssystem. Das Wissen über den Zusammenhang zwischen Rauchen und Tuberkulose kann genutzt werden, um ein Programm zur Tuberkuloseprävention und -kontrolle zu entwickeln.

**Methode:**

Es handelt sich um eine retrospektive Beobachtungsstudie. Über einen Zeitraum von 18 Monaten wurden 100 Patienten mit Sputumabstrich positiver Lungentuberkulose im Rahmen des überarbeiteten nationalen Tuberkulose-Kontrollprogramms (RNTCP) analysiert.

**Ergebnisse:**

1+ Sputum-Positivität wurde bei einer höheren Anzahl von Nichtrauchern (77%) beobachtet, gefolgt von Ex-Rauchern (43%) und Rauchern (2%). 2+ bzw. 3+ Sputum-Positivität wurde bei einer höheren Anzahl von Rauchern (63% bzw. 35%) beobachtet, gefolgt von Ex-Rauchern (29%) und Nicht-Rauchern (18% bzw. 5%).

**Schlussfolgerung:**

Raucher wiesen im Vergleich zu Nichtrauchern ausgedehnte Infiltrate auf. Außerdem nahm mit dem Schweregrad des Rauchens (Raucherindex) auch die bakterielle Belastung zu (höhere Grade der Sputumpositivität). Raucher hatten auch ein schlechteres Ergebnis in Bezug auf den Behandlungserfolg als Nichtraucher.

## Introduction

Tobacco smoking and tuberculosis (TB) are the two major health problems, especially in developing countries. The World Health Organization (WHO) declared tuberculosis a ‘global emergency’ [1]. While some people consider TB to be a “dead” disease that was eradicated years ago, it is in fact very much alive in the world. In developing countries like India, it poses a tremendous burden on healthcare facilities [2], [3]. Smoking is unfortunately seen as socially acceptable, despite it being a major health hazard [4]. The socio-economic conditions, including poverty, over-crowding, poorly ventilated rooms with no natural light, poor nutrition, and alcohol abuse have been associated with smoking and are also known risk factors for tuberculosis infection [5]. Potentially, smoking is one of the most modifiable exposures, but smoking alone as a contributor to morbidity and mortality due to tuberculosis has not been very well validated [5]. Importantly, it is not only the smoker who is at risk of tuberculosis; studies have also proven the role of passive smoking, secondhand smoke, and environmental tobacco smoke exposure (ETS) as contributory factors for active tuberculosis [6]. Previous studies showed an association between smoking, alcohol, and the increased risk of pulmonary tuberculosis (PTB) [7], [8], [9]. Smoking may weaken the immune system by disturbing the function of epithelial permeability, cilia movement, and macrophages [10]. Despite the fact, that smoking is one of the factors cited in the literature, the association between smoking and PTB has been assessed in very few studies. Using WHO guidelines, there are effective TB control programs and highly effective treatment is available. However, if people are not aware of its seriousness and preventability, especially in terms of the behavioral risk factors, it will remain a public health problem in India. For these reasons, this study examined cigarette smoking as a risk factor associated with PTB manifestation in adults. This knowledge can be utilized to develop a program for TB prevention and control.

## Method

This is a retrospective observational study over 18 months involving 100 diagnosed cases of sputum smear-positive pulmonary tuberculosis in patients treated under the Revised National Tuberculosis Control Program (RNTCP). Seth GS Medical College and KEM Hospital Mumbai Maharashtra India Institutional ethics committee approval was given. Details were obtained from the clinical history proforma, and patient details were recorded by the Directly Observed Treatment Short course (DOTS) care provider in the tertiary care center at Seth GS Medical College and KEM Hospital Mumbai Maharashtra India.

### Sample size

100 sputum smear-positive tuberculosis patients being treated under RNTCP were included. The average yearly follow-up rate of sputum-positive patients is 100–120 patients per year [5], [10] at Seth GS Medical College and KEM Hospital Mumbai Maharashtra India, hence a sample size of 100 was representative.

### Inclusion criteria

Sputum smear-positive pulmonary tuberculosis patients above 18 years of age whose data – including addiction history – were available in the records (inpatient and with the DOTs, who were previously treated, were treatment-naive or who completed treatment).

### Exclusion criteria

Age below 18 years and sputum smear-negative pulmonary and extrapulmonary tuberculosis.

### Analyzed data

All sputum smear-positive pulmonary tuberculosis patients under the RNTCP were studied and a retrospective analysis of their addiction to smoking was conducted, for which details were obtained from clinical history proforma (filled out previously for admitted patients) and patient details recorded by the DOTS care provider in the tertiary care center.

Smokers were classified as smokers, ex-smokers and nonsmokers. 

The outcome of each participant was analyzed with respect to:

1. Clinical symptoms of TB, such as cough, hemoptysis, fever, loss of weight and appetite
2. Acid-fast bacteria (AFB) smear test: Bacterial load as determined by microscopy;
  1+=10–99 AFB (measure of viable colonogenic cell numbers in CFU/MI) in the visual fields are examined from one end to another, 2+=1–10 AFB per field in at least 50 visual fields, and 3+=>10 AFB per field in at least 20 visual fields
3. Radiological signs, e.g., like cavitary or non-cavitary lesions (these are radiological terms for tubercular pathology), unilateral/bilateral lesions (either one lung or both lungs involvement) (upper zone/middle zone/lower zone)
4. Treatment outcome is divided into favorable – i.e., the patient is cured completely – and unfavorable ones, e.g., defaulters, failed, multidrug-resistant (MDR), death. All the above categories are as per RNTCP definition:

– Cured: Patient whose sputum smear or culture was positive at the beginning of treatment but who was smear- or culture-negative in the last month of treatment and on at least one previous occasion.

– Defaulter: Patient whose treatment was interrupted for 2 consecutive months or more without medical approval.

– Failed: A patient whose sputum smear or culture is positive at 5 months or later during treatment. Also included in this definition are patients found to – harbor a multidrug-resistant (MDR) strain at any point of time during the treatment, whether they are smear-negative or smear-positive.

– Dead: Patient who dies for any reason during treatment.

### Smoking exposure

The *pack-year* is a unit for measuring the amount a person has smoked over a long period of time. It is calculated by multiplying the number of packs of cigarettes smoked per day by the number of years the person has smoked. For example, 1 pack-year is equal to smoking 20 cigarettes (1 pack) per day for 1 year, 40 cigarettes per day for half a year, and so on. One pack-year is the equivalent of 365.24 packs of cigarettes or 7,305 cigarettes [11].

Quantification of smoking was done using the *smoking index* (SI). As previously published, SI was defined as the number of *bidis*/cigarettes smoked per day multiplied by the number of years smoked [12], [13]. The concept of using SI for quantification of smoke exposure is here based on *bidi* – the hand-rolled form of tobacco wrapped in the dried tendu leaf – as it is the most common smok-ing product in India [14]. Moreover, the number of *bidis* in a given pack is variable in contrast to cigarettes, since the former is a cottage industry with much less standardization in its manufacturing process. It has been shown in previous studies that *bidis* and cigarettes are associated with similar risks in relation to lung cancer and that for calculating time-intensity tobacco smoke exposure, one *bidi* should be equivalent to one cigarette [15], [16], [17]. 

### Statistical analysis

All collected data was entered into a Microsoft Excel sheet and then transferred to SPSS software ver.22 for analysis. Qualitative data was presented as frequency and percentages, and analyzed using chi-squared test. A P-value <0.05 was considered statistically significant. 

## Results

Most of the study population were smokers (49%), followed by nonsmokers (44%) and ex-smokers (7%). The AFB load in sputum was higher in smokers than in ex-smokers and nonsmokers (Table 1 (Tab. 1)). 

Radiographs showed non-cavitary lesions in 56% and cavitary lesions in 44% of patients, respectively (Table 2 (Tab. 2)).

Complete cure was observed in the highest number of nonsmokers (93%), followed by ex-smokers (86%) and smokers (65%). Defaulters were most frequent among smokers (31%), followed by ex-smokers (14%) and nonsmokers (7%). Failed treatment was observed in higher numbers of smokers (2%) vs ex-smokers (0%) and nonsmokers (0%). Probable MDR was observed in a higher number of smokers (2%) compared to ex-smokers (0%) and nonsmokers (0%), with no mortality in any patients (Table 3 (Tab. 3)).

## Discussion

Smoking has been consistently linked with TB in recent years, and its increased risk among smokers may be explained by the effect of smoking on pulmonary host defenses. Chronic exposure to tobacco, as well as to several environmental pollutants, impairs the normal clearance of secretion on the trachea-bronchial mucosal surface and may thus allow the causative organism *Mycobacterium (M.) tuberculosis*, to escape the first level of host defenses which prevent the bacteria from reaching the alveoli [18]. Smoke also impairs the function of pulmonary alveolar surfactants [19]. Recent works have suggested a novel mechanism: Nicotine is hypothesized to act directly on nicotine acetylcholine receptors on macrophages to decrease the production of intracellular tumor necrosis factor and thus impair the killing of *M. tuberculosis* [20]. These effects of smoking on pulmonary host defense support a causal link between smoke exposure and either an increased risk of acquiring TB or progression of TB. Several studies have shown an association between smoking and TB-related infection, disease and increased mortality [21], [22], [23], [24], [25]. A recent meta-analysis reported that those who had close contact with a smoking household member were 9 times more likely to have TB as compared to those who had distant contacts [26]. Although the current approach to TB control focuses on case detection and treatment, recent studies suggest that this strategy alone might not be sufficient to achieve this goal, and it may also be necessary to reduce risk factors that contribute to the occurrence of TB infection and/or disease. Such risk factors may act at one of several steps in the natural history of the disease [21]. Tobacco-attributable deaths are projected to increase from 3 million in 1990 to 8.4 million in 2020 [27]. This harmful socioeconomic factor has in the past neglected. A higher bacterial load was found in patients with a history of smoking. However, due to a smaller sample, this difference, when compared to nonsmokers, could not reach statistical significance as found in other studies [22]. A sputum-smear grade of 3+ was more common among moderate and heavy smokers than those with a SI of <100, which was also observed by Rathee et al. [28]. The higher grades of sputum smear-AFB positivity could possibly be explained by the fact that cavitation and advanced infiltrates are more common among smokers. These findings agreed with the study by Rathee et al. [28], in which smokers had a significantly (P=0.001) lower treatment success rate (69%) than did nonsmokers and former smokers (93.8% and 90.9%, respectively). However, it was also observed that the default rate was higher among smokers (12/42, 28.5%) than former smokers and nonsmokers (9.1% and 6.3%, respectively, P=0.001). A higher default rate among smokers was largely responsible for the poor treatment success among them [28].

## Conclusion

Smoking and TB both affect the younger age group of the population, with male predominance. Smokers demonstrate extensive infiltrates as compared to nonsmokers. Furthermore, as the severity of smoking increased (smoking index), the bacterial load also increased (higher grades of sputum positivity). At the end of the treatment, smokers exhibited more radiological sequelae in terms of cavitation. Smokers also had a poorer outcome in terms of treatment success rate as compared to nonsmokers. However, this was largely due to the high default rate among smokers, implying compliance or treatment-adherence issues among smokers as a main barrier to treatment success. There is an urgent need for default retrieval action among this subgroup of TB patients, including motivation and counselling. TB control programs may also benefit from a focus on smoking cessation interventions. 

## Notes

### Authors’ ORCIDs

- Mahesh Shinde: 0000-0002-4091-9447
- Sangramsingh Dixit: 0000-0002-2411-9108
- Mihir Patel: 0000-0001-6304-5845
- Atharva Sharma: 0009-0005-9365-0882
- Juily Satam: 0009-0008-7201-0714
- Yogeshwari Patil: 0009-0006-4326-6222
- Dheer Upadhyay: 0009-0006-0033-1856
- Shreyasi Chiwadshetti: 0009-0008-2292-3312
- Adhiraj Mathur: 0009-0006-9768-1448
- Varad Rege: 0009-0002-3548-1337
- Arunojya Kumari: 0009-0005-1877-7816
- Hiya Seth: 0009-0005-8274-8294

### Ethical approval 

Seth GS Medical College and KEM Hospital Mumbai Maharashtra India Institutional ethics committee approval was given (Registration no. ECR/417/Inst./MH/2013/RR-19).

### Funding 

None. 

### Competing interests

The authors declare that they have no competing interests. 

## Figures and Tables

**Table 1:**
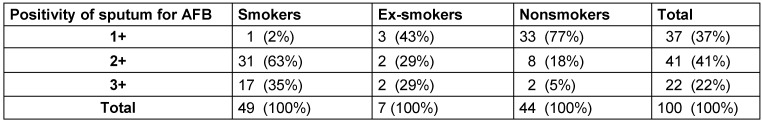
Positivity of sputum for AFB depending on smoking history

**Table 2:**
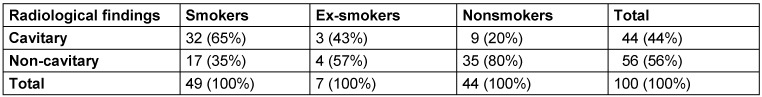
Radiological findings

**Table 3:**
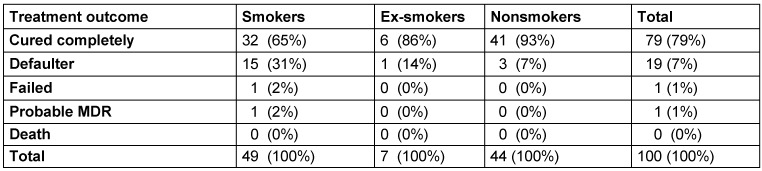
Treatment outcome depending on smoking history
